# Atrial fibrillation recurrence risk after single catheter ablation in patients with history of hyperthyroidism: systematic review and meta-analysis

**DOI:** 10.1186/s43044-025-00696-2

**Published:** 2025-10-30

**Authors:** Rivaldo Rivaldo, Kevin Tjoa, Peter Parulian, Michael Sugiyanto, Henrico Catrawijaya, Birry Karim

**Affiliations:** 1 Faculty of Medicine, Universitas Indonesia, Jl. Salemba Raya No. 6, Jakarta, 10430 Indonesia; 2 Cardiology Division, Internal Medicine Department, Faculty of Medicine Universitas, Indonesia-Cipto Mangunkusumo Hospital, Jakarta, Indonesia

**Keywords:** Atrial fibrillation, Hyperthyroidism, Catheter ablation, Recurrence

## Abstract

**Background:**

Atrial fibrillation (AF) is the most common arrhythmias. Other than pharmacotherapy, catheter ablation is preferred especially for symptomatic paroxysmal AF or persistent AF. However, recurrence of atrial fibrillation following catheter ablation can occur due to several factors. Hyperthyroidism is known as a factor in atrial fibrillation pathogenesis but its role in AF recurrence following ablation is not known yet. Therefore, we aimed to assess the recurrence risk after catheter ablation in patients with a history of hyperthyroidism.

**Methods:**

Systematic searching was performed through three databases: MEDLINE, EMBASE, and SCOPUS for studies reporting the recurrence of AF (hazard ratio) following catheter ablation in patients with a history of hyperthyroidism. The risk of bias assessment of included studies was performed using quality in prognosis studies (QUIPS). Meta-analysis (random effect model and inverse variance) was conducted using RevMan software version 5.4.

**Results:**

Four studies involving 837 subjects were included. Pooled analysis shows a higher risk of recurrence of atrial fibrillation following catheter ablation in patients with history of hyperthyroidism (HR 1.86 [CI 95% 1.26–2.75]; *I*^2^ 38%). Subgroup analysis showed patients with amiodarone-induced hyperthyroidism (AIH) had a higher risk of atrial fibrillation recurrence (HR 2.31; CI 95% 1.49–3.58; *I*^2^ 0%) compared to non-AIH. However, two studies on AIH showed moderate risk of bias.

**Conclusions:**

History of hyperthyroidism was found as the risk of recurrence of atrial fibrillation after a single ablation procedure. Patients with amiodarone-induced hyperthyroidism have a higher recurrence risk. Further studies with larger participants are needed for subgroup analysis on specific parameters of the ablation.

**Supplementary Information:**

The online version contains supplementary material available at 10.1186/s43044-025-00696-2.

## Background

Atrial fibrillation (AF) has long been recognized as the most common arrhythmia worldwide, with an estimated global prevalence of approximately 37.57 million cases (0.51%) in 2017 [11]. This prevalence may rise to 3–4% when asymptomatic cases are included [1]. Over the past two decades, the prevalence of AF has increased by up to 33% [11]. Sociodemographic and regional disparities have been observed, with countries of higher sociodemographic index showing a greater prevalence and risk of AF [15]. AF poses a significant burden on clinical outcomes and healthcare systems, evidenced by an increase in cardiovascular-related mortality from 18 per 100,000 population in 2011 to 22.3 per 100,000 in 2018 [7]. These trends highlight ongoing challenges in AF management, even in high-income countries.

Rate and rhythm control remain the cornerstone of AF therapy, with demonstrated benefits in symptom relief and reduction of major cardiovascular events such as heart failure and stroke [9]. However, long-term pharmacotherapy can be burdensome, and the failure rate of medical therapy, even when optimized, ranges from 30 to 46% [16]. In patients with symptomatic paroxysmal (PAF or persistent AF (PeAF who do not respond to antiarrhythmic medications, catheter ablation is considered the treatment of choice. Studies have shown that catheter ablation not only improves symptoms through rhythm control but also reduces AF recurrence [5, 18]. Nevertheless, recurrence remains a significant issue following catheter ablation, with rates reaching up to 40% within the first year, excluding the blanking period [8]. Despite this, catheter ablation still demonstrates superior outcomes compared to medical therapy, which may have recurrence rates as high as 77% within one year [6].

Multiple factors have been associated with AF recurrence, regardless of treatment modality. These include female sex, advanced age, and hypertension. In patients undergoing catheter ablation, additional risk factors include the presence of cardiovascular disease, multiple AF foci (especially those originating from the left atrial free wall), and left atrial enlargement. While hyperthyroidism has been implicated in the pathogenesis of AF [4, 19, 20], its role in AF recurrence after catheter ablation remains unclear. This study aims to evaluate the risk of AF recurrence following catheter ablation in patients with hyperthyroidism.

## Methods

### Searching strategies

Systematic searching and reporting were conducted in accordance with the Cochrane Handbook for Systematic Reviews and the Preferred Reporting Items for Systematic Reviews and Meta-Analyses (PRISMA) guidelines. Comprehensive literature searches were performed in PubMed, EMBASE, and Scopus by three independent investigators (KT, R, and MS) up to April 2023, without restrictions on publication date or language. The complete search strategies are provided in **Supplementary Material S1**. In addition, reference lists of relevant articles were hand-searched to identify any additional studies. This review was registered in **PROSPERO** (CRD42023453167).

### Study eligibility criteria

Original studies evaluating the risk of atrial fibrillation (AF) recurrence after catheter ablation in patients with a history of hyperthyroidism, compared to those without such a history, were included if they met all of the following criteria: (1) the hazard ratio (HR) was reported or could be calculated, (2) the study population consisted of adult patients (aged > 18 years), and (3) the ablation procedure was performed during a euthyroid state. Studies were excluded if they were non-original (e.g., reviews, editorials), lacked full-text availability, or were not published in English.

### Data extraction and outcomes recording

Data extraction was performed independently by three investigators (R, MS, PPH) and verified by a fourth investigator (KT). Predefined data elements included study design, study location, number of subjects, age, type of ablation, etiology, follow-up duration, recurrence rate, time to recurrence or recurrence-free survival, and hazard ratio (HR) with 95% confidence intervals (CI). These data were recorded in a prespecified table. Potential confounding variables—such as duration of hyperthyroidism, use of medications, and number of ablation procedures—were also extracted when reported. For studies that provided only Kaplan–Meier survival curves, HRs were estimated using the IPDfromKM tool (available at: https://www.trialdesign.org/one-page-shell.html#IPDfromKM). When available, adjusted HRs at the end of follow-up were used in the meta-analysis. In cases of overlapping data across studies, the study with the largest sample size was included.

### Risk of bias assessment

Quality in prognosis studies (QUIPS) was used for risk of bias (RoB) assessment. RoB assessment was done by three independent investigators (KT, R, PP), and any discrepancies were solved through discussion to reach consensus. When no consensus was reached, a decision was made in consultation with the fourth investigator (BK).

### Statistical analysis

Meta-analysis was performed where appropriate using RevMan 5.4. A random-effects model with inverse variance weighting was applied to calculate pooled estimates, given the anticipated heterogeneity across studies. Both adjusted and unadjusted effect estimates were analyzed; however, only adjusted estimates were considered for the primary interpretation of results. Heterogeneity was assessed using the *I*^2^ statistic and categorized as negligible (0–25%), low (25–50%), moderate (50–75%), or high (> 75%). Publication bias was assessed using funnel plots, as well as Egger’s and Begg’s tests when at least ten studies were available. Pre-specified subgroup analyses were conducted based on (1) etiology, (2) geographical location, (3) type of ablation, and (4) RoB assessment.

## Results

### Studies characteristics and outcomes

**Fig. 1:**
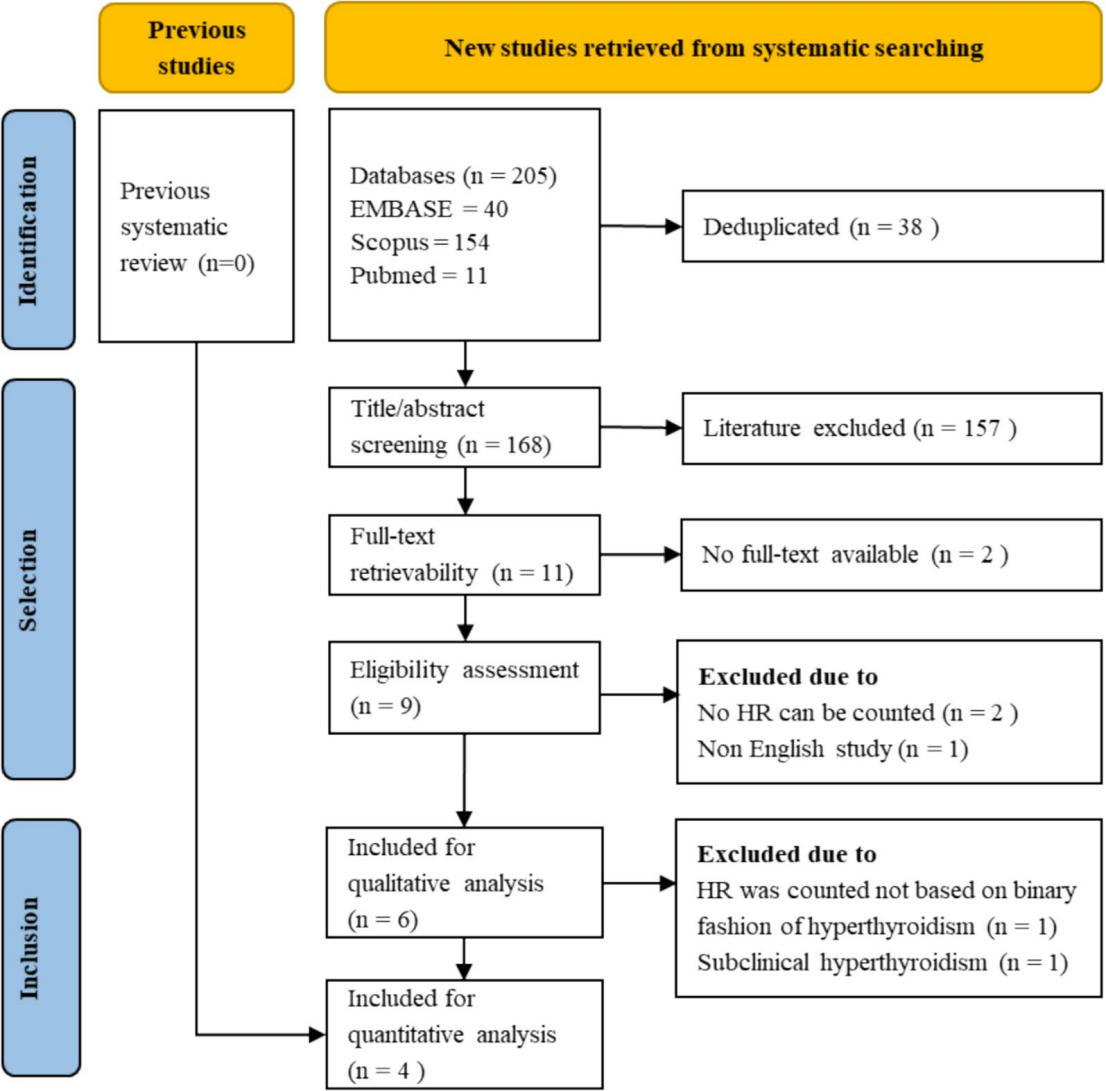
PRISMA flowchart

Out of 205 studies identified through systematic searching, four studies (*n *= 837) were included in the quantitative analysis [13, 14, 21, 22]. The study selection process is detailed in Fig. 1. Three studies were prospective cohort designs, and one was a case–control study. Three studies were conducted in Asian populations, while one study was conducted in a Western population (Russia). The reported age of participants ranged from 56 to 62 years, with a predominance of male participants across most studies. All studies employed circumferential pulmonary vein isolation as the primary ablation technique. The follow-up duration ranged from 12 to 48 months.

Regarding the reported outcomes related to recurrence, Mikhaylov et al. [14] and Wang et al. [21] separated early and late recurrence (both were specific for AIH). However, both are not specifically mentioned AF, yet any tachyarrhytmias. Early recurrence is defined as the recurrence of AF within 3 months after the initial ablation. On the other hand, the other two studies reported specific AF recurrence [13, 22]. The recurrence rate across studies ranged from 26.6 to 54%, with an overall recurrence rate of 42.5% (*n* = 356). Patients with a history of hyperthyroidism exhibited a higher recurrence rate (55.47%) compared to those without (40%). However, none of the included studies reported time to recurrence or recurrence-free survival. Reported hazard ratios (HRs) ranged from 0.87 to 3.32, with only one study [13] reporting no significant increase in recurrence risk. The highest HR was observed in a study focusing on early AF recurrence. Table 1 summarizes the study characteristics and reported outcomes. Additional details on treatment regimens and adjusted covariates are provided in **Supplementary Material S2.**

**Table 1:** Studies’ characteristics and outcomes

No	Author, year	Study design; location	Subject	AF duration (HTH versus NHTH)	Types of AF	Etiology	Euthyroid period (months)	Primary ablation	Follow-up (months)	Recurrence		HTH	NHTH	HR [CI95%]
			N; age; %male							Reported	%total	R versus NR	R versus NR	
1	Machino, 2012	Prospective cohort; Japan	337; 61; 80.7	60 ± 36 versus 72 ± 72	PAF (57.3%), peAF (42.7%)	100% Graves’ disease	3	CPVI	48 ± 12	Any AF, overall	43.3	7 versus 9	139 versus 182	0.87 [0.4–1.88]*
2	Mikhaylov, 2013	Case– control; Russia	60; 58.6; 55	73.9 ± 35.9 versus 77.2 ± 29.6	PAF	100% AIH	3	CPVI	12	Any TA, early and overall	48.3	14 versus 6	15 versus 25	Early: 3.28 [1.36–7.87] Overall: 2.45 [1.35–4.45]
3	Wanwarang, 2015	Prospective cohort; Taiwan^a^	390; 56; 51	NA	PAF (75%), peAF (25%)	46% Graves’ disease; 26% thyroiditis; 28% AIH	3	CPVI	31 ± 14	Any AF, overall	39.4	40 versus 41	114 versus 195	2.07 [1.27–3.38]*
4	Wang, 2016	Prospective cohort; China	50; 61.9; 46.6	12.75 ± 13.23 versus 20.07 ± 34.29	PAF	100% AIH	NA	CPVI	12	Any TA, early and overall	54	15 versus 5	12 versus 18	Early: 3.32 [1.42–7.73] Overall: 2.15 [1.12–4.13]

AF: atrial fibrillation; AIH: amiodarone-induced hyperthyroidism; CI: confidence interval; CPVI: circumferential pulmonary vein isolation; CTIA: cavotricuspid isthmus ablation; HR: hazard ratio; HTH: hyperthyroidism; NA: not applicable; NHTH: no hyperthyroidism; NR: no recurrence; R: recurrence; RFCA: radiofrequency catheter ablation; PAF: paroxysmal AF; peAF: persistent AF; TA: tachyarrhythmia

^a^Data from matched cohort after propensity score matching for age, gender, hypertension, diabetes mellitus, congestive heart failure, coronary artery disease,and previous stroke or transient ischemic attack, while the original cohort consisted of 719 participants (633 without HTH, 84 with HTH)

^*^Adjusted HR

### Pooled estimates

**Fig. 2:**
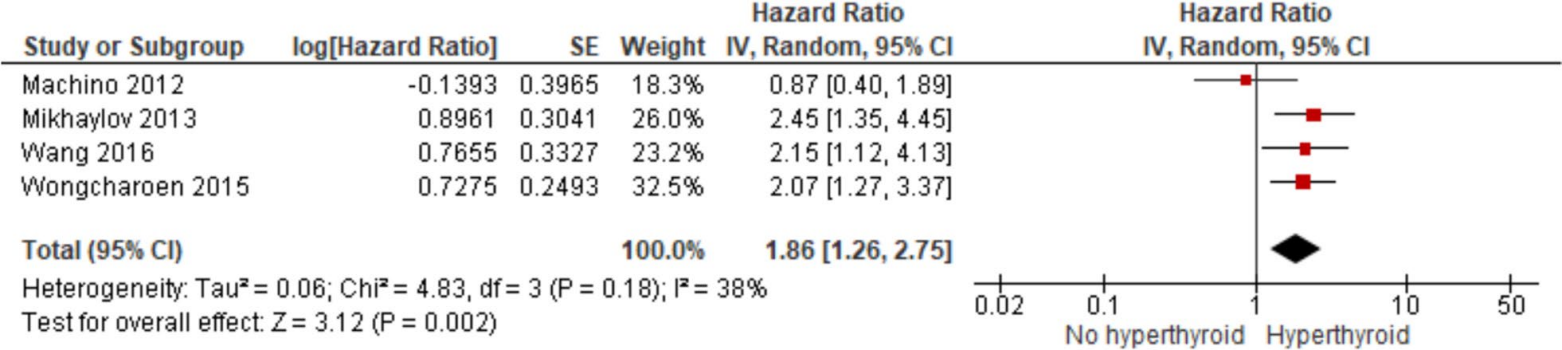
Overall pooled estimates on the risk of AF recurrence on hyperthyroidism

**Fig. 3:**
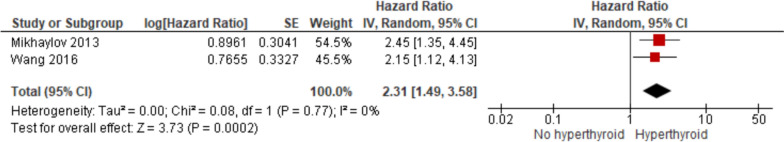
Subgroup analysis on risk of overall AF recurrence on amiodarone-induced hyperthyroidism

**Fig. 4:**
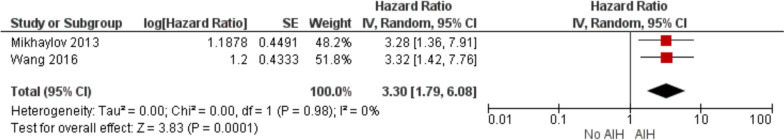
Subgroup analysis on risk of early AF recurrence on amiodarone-induced hyperthyroidism

The pooled risk of atrial fibrillation (AF) recurrence after a single catheter ablation procedure in patients with hyperthyroidism was significantly elevated, with a hazard ratio (HR) of 1.86 (95% CI 1.26–2.75) and low heterogeneity (*I*^2^ = 38%) (Fig. 2). Subgroup analysis by etiology revealed that amiodarone-induced hyperthyroidism (AIH), as reported by Mikhaylov et al. [14] and Wang et al. [21], was associated with a higher recurrence risk (HR: 2.31,95% CI 1.49–3.58; *I*^2^ = 0%) (Fig. 3), compared to non-amiodarone etiologies such as Graves’ disease, as shown in the study by Machino et al. [13] (HR: 1.15,95% CI 0.53–2.50). In contrast, Wongcharoen et al. [22], who included patients with various etiologies of hyperthyroidism, reported a moderately elevated recurrence risk (HR: 2.07,95% CI 1.27–3.37). Among the included studies, only two (Machino et al. and Wongcharoen et al.) reported adjusted hazard ratios, whereas the two studies focusing on AIH (Mikhaylov et al. and Wang et al.) did not adjust for potential confounders. Notably, both AIH studies reported outcomes stratified by early recurrence (defined as recurrence within 3 months post-ablation), demonstrating a higher risk of early recurrence among hyperthyroid patients (Fig. 4). However, since this finding is based solely on the AIH subgroup, its generalizability to other etiologies of hyperthyroidism remains limited.

### Risk of bias assessment

All studies were assessed for RoB using QUIPS. Two studies on AIH population had moderate risk of bias that arose from the confounding and analysis/reporting domains. Wang et al. [21] did not report the hazard ratio and survival curve although provided survival table summary. The study also had limited statistical adjustment although recorded confounders were observed. Mikhaylov et al. [14] did Cox analysis and adjustment yet no hazard ratio was reported.

**Fig. 5:**
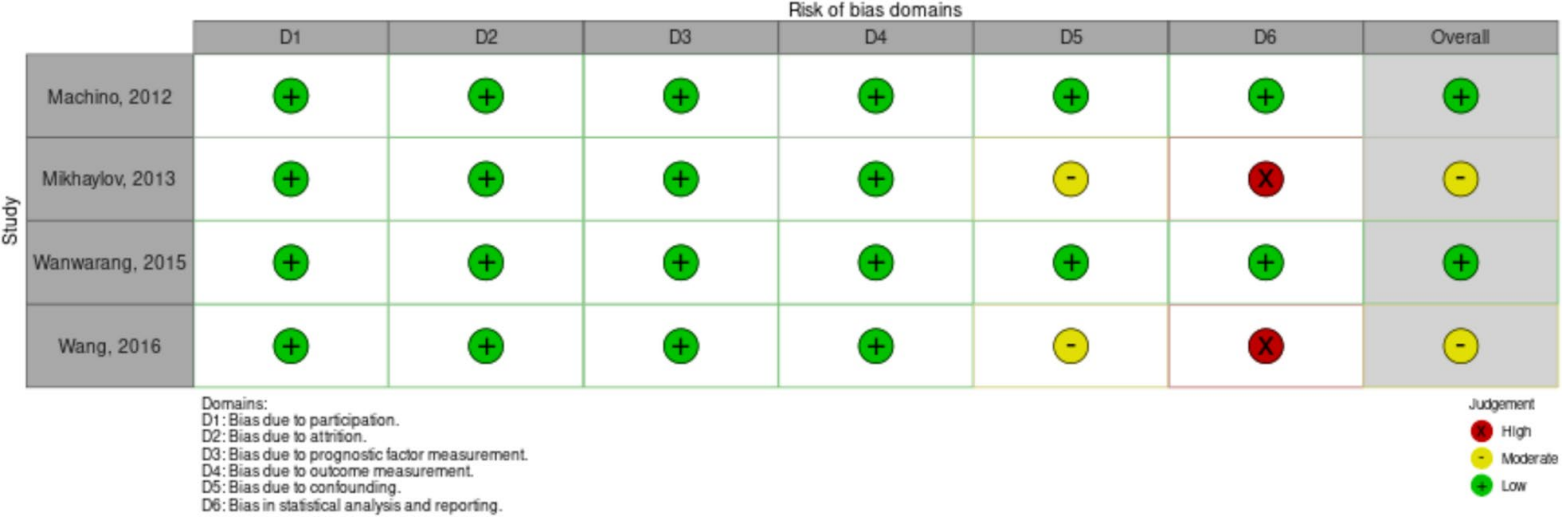
Risk of bias assessment on individual study

**Fig. 6:**
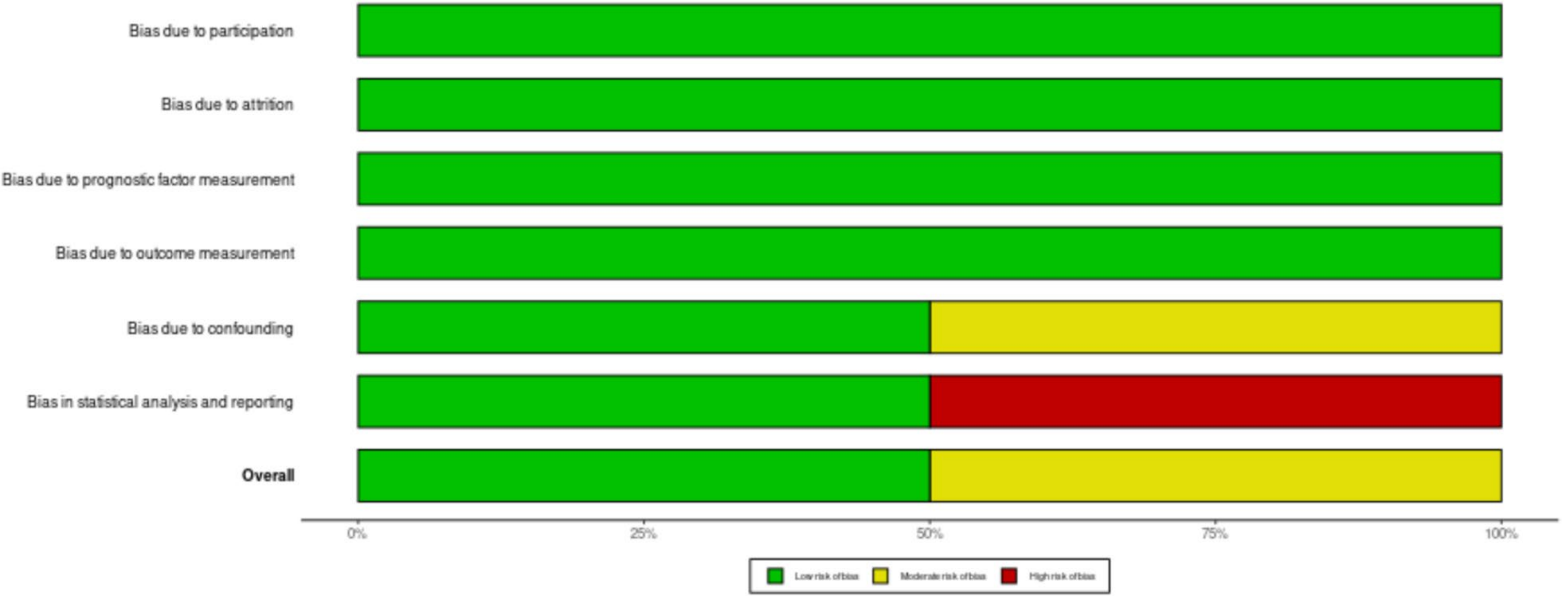
Summary of RoB assessment on each domain

On the other hand, the other two studies gave sufficient statistical analysis and adjustment through multivariate analysis or subjects matching. No adjustment on possible covariates, especially in studies about AIH. Figs. 5, 6 show the RoB plot for individual studies and the summary for each domain, respectively. No publication bias assessment was conducted due to a limited number of studies.

## Discussion

### AF recurrence and hyperthyroidism

Our study found that history of hyperthyroidism increased the risk of AF recurrence after ablation procedure. Despite various etiology (AIH or no AIH), risk of AF recurrence consistently elevated in patients with a history of hyperthyroidism. To our knowledge, no other systematic review and meta-analysis has addressed this issue. Thus, this study is the first study to estimate the pooled risk of AF recurrence after ablation procedure in patients with history of hyperthyroidism.

The observed increase in recurrence risk is supported by established pathophysiological mechanisms. Hyperthyroidism promotes structural and electrophysiological remodeling of cardiomyocytes through changes in gene expression and intracellular signaling pathways, including upregulation of PI3K, MAPK, and Akt pathways. These changes enhance *β*-adrenergic receptor expression and Na^+^/K^+^ ATPase activity, contributing to a pro-arrhythmic substrate. Chronic hyperthyroidism can also lead to ventricular wall thickening, increased contractility, enhanced relaxation velocity, and alterations in ion channel expression and intracellular calcium handling—changes that mirror those seen in heart failure [3, 2, 17]. The degree of structural and molecular changes may impact the time to arrhythmia recurrence. Unfortunately, in our review, we only found the distinction between early and late recurrence from studies on AIH by Mikhaylov et al. [14] and Wang et al. [21] that should be confirmed in further studies.

Subgroup analyses revealed that patients with AIH had the highest risk of AF recurrence ([HR 2.31; 95% CI 1.49–3.58]), followed by those with all-cause hyperthyroidism ([HR 2.07; 95% CI 1.27–3.37]) and Graves’ disease-related hyperthyroidism ([HR 1.15; 95% CI 0.53–2.50]). However, these comparisons should be interpreted cautiously. For instance, the study by Wongcharoen et al. [22], which reported on all-cause hyperthyroidism, included a mixed population of 28% AIH, 46% Graves’ disease, and 26% thyroiditis. Furthermore, none of the included studies directly compared different etiologies of hyperthyroidism (e.g., AIH vs. Graves’ disease), as all comparisons were made against control groups without a history of hyperthyroidism.

Current European Society of Cardiology (ESC) guidelines recommend amiodarone as a last-line therapy for rate control in AF, reserved for cases where *β*-blockers, non-dihydropyridine calcium channel blockers, or digoxin fail [5]. It is therefore plausible that patients with AF managed with amiodarone represent a population with more persistent, long-standing persistent, or permanent AF phenotypes. Notably, even in this context, the efficacy of amiodarone remains modest, with success rates around 40% [12]. Consequently, the elevated recurrence risk observed in AIH should not be overgeneralized to all hyperthyroid populations, and further studies comparing AIH and non-AIH directly are needed to clarify these findings.

From the perspective of electrophysiological parameters, particularly regarding arrhythmic foci targeted during ablation, only two studies [21, 22] reported significant baseline differences between patients with and without a history of hyperthyroidism. Both studies consistently identified a higher prevalence of non-pulmonary vein (non-PV) ectopic foci among hyperthyroid patients (40–50% versus 6–23%), which is relevant since these ectopic sources may require additional ablation beyond standard PV isolation. However, Machino et al. [13] reported no significant differences in the need for additional ablation between groups, and Mikhaylov et al. [14] did not report on ectopic foci or ablation strategy. Importantly, none of these studies explored the direct association between the presence of ectopic foci or the extent of additional ablation and AF recurrence, as these were reported only as baseline characteristics.

Regarding recurrence risk, Machino et al. [13] found that a history of hyperthyroidism was not significantly associated with AF recurrence and was not an independent predictor in multivariate analysis (HR: 0.87,95% CI 0.40–1.88). A key methodological difference in their study was the inclusion of both paroxysmal and persistent AF (peAF), with 42.7% of their cohort having peAF—substantially higher than the proportion reported by Wang et al. [21] (23.8%). Since peAF is an established independent risk factor for recurrence post-ablation, its inclusion may have confounded the effect of hyperthyroidism on outcomes. In contrast, Wongcharoen et al. [22] and Mikhaylov et al. [14] exclusively enrolled patients with paroxysmal AF (PAF), allowing for a more homogeneous assessment.

Although this review did not focus on outcomes following repeat ablation, it is notable that Wongcharoen et al. [22] conducted follow-up on patients who underwent a second procedure due to recurrence. In this subset, hyperthyroidism history was not associated with further recurrence, suggesting that certain elements of thyroid hormone-induced cardiac remodeling may be partially reversible after euthyroid restoration. However, persistent AF after euthyroidism remains common, affecting up to 33% of patients [12, 13]. This supports the hypothesis that structural, rather than hormonal, mechanisms may be central to long-term recurrence risk. Machino et al. [13] identified left atrial (LA) diameter as a significant predictor of recurrence, while hyperthyroidism history was not. Similarly, Wongcharoen et al. [22] found that both LA diameter and mean LA voltage were associated with recurrence, reinforcing the role of atrial remodeling in post-ablation outcomes. The potential time-dependent effects of thyroid hormone restoration also warrant consideration. While hormonal correction may reduce short-term risk, long-term molecular and structural changes, including fibrosis and ion channel remodeling, may persist. Factors such as age, comorbidities, race, and treatment history may modify this trajectory [17].

Furthermore, a study by Li et al. [10] broadened the spectrum of thyroid dysfunction by including subclinical states. They found that subclinical hyperthyroidism—but not hypothyroidism—was associated with a higher risk of AF recurrence compared to euthyroid patients. Although subgroup analyses (e.g., by age, sex, and AF type) yielded no statistically significant differences, restricted cubic spline regression models demonstrated no linear association between recurrence risk and serum levels of TSH, free T4 (fT4), or free T3 (fT3). These findings raise important questions about the existence of a dose–response relationship between thyroid hormone levels and recurrence risk, suggesting a need for further investigation.

### Heterogeneity and generalization

Our study stipulates no important heterogeneity observed on our analysis, either overall pooled risk estimates (*I*^2^ = 32%; *p* = 0.22) or subgroup analysis of AIH (*I*^2^ = 0%; *p* = 0.98). However, careful interpretation of results is needed, especially for patients with AIH since no statistical adjustment attempt was done from both available studies [14, 21]. Although baseline sociodemographic characteristics were mostly similar, several studies showed that certain parameters related to electrophysiological profiles (i.e., number of arrhythmic foci, heart chamber voltage) were not addressed in all studies. When electrophysiological profiles were reported, the limited number of studies (only two studies) [13, 22] and different categorization/measurement of variable (e.g., LA diameter reported in increment per mm and per 10 mm) prevent further subgroup analysis. See **Supplementary material S2** for adjusted variables on each study. A massive attempt on controlling heterogeneity that arises from sociodemographic profiles using propensity score matching was done despite only being available in a single study [22].

### Limitations

Although none of the included studies exhibited a high risk of bias and all were derived from prospective inception cohort designs, several limitations of this review should be acknowledged. None of the studies explicitly specified the class of atrial fibrillation (AF) enrolled (i.e., paroxysmal, persistent, or long-standing persistent), which limits the interpretability and applicability of the findings. While radiofrequency ablation is generally accepted as a treatment option across the AF spectrum—including persistent and permanent forms—differences in recurrence risk between AF subtypes have been observed. For instance, Li et al. [10] demonstrated differential recurrence risks between paroxysmal and persistent AF, suggesting that AF subtype stratification is crucial for accurate prognostication.

We found no included studies that reported median time to recurrence or recurrence-free survival (RFS), which hinders the ability to assess the temporal dynamics of AF recurrence and to provide a detailed prognosis over time. This gap in time-to-event data reduces the clinical utility of the pooled estimates, especially in guiding patient expectations and follow-up strategies.

Furthermore, this review could not elucidate a dose–response relationship between thyroid hormone levels and AF recurrence. The absence of detailed thyroid function data and limited adjustment for confounding factors in some studies—particularly those investigating amiodarone-induced hyperthyroidism (AIH)—constrains causal interpretation. Furthermore, the classification of recurrence as early or late was reported only in studies on AIH, emphasizing a lack of uniformity in outcome definitions across etiologies of hyperthyroidism. This highlights the need for future studies to stratify patients based on the underlying cause of thyroid dysfunction, as different etiologies (e.g., Graves’ disease vs. AIH) may confer distinct arrhythmic risk profiles.

Finally, our literature search was restricted to English-language publications. As a result, potentially relevant studies published in other languages may have been missed, introducing the possibility of language bias in this review.

### Recommendations

The history of hyperthyroidism is important to address during AF management since even subclinical hyperthyroidism increases the risk of AF recurrence. Patients should be in a euthyroid state at least 3 months before ablation. We recommend that every AF patient needs to be screened for hyperthyroidism. Routine monitoring of thyroid hormone level should be conducted on AF patients treated with amiodarone and also should be examined before and after ablation procedure to determine the thyroid status of the patients. AF patients that are proven to have hyperthyroidism may require endocrinology consultation to achieve satisfactory control. Further larger studies that enable subgroup analysis on ablation specific parameters (i.e., baseline electrophysiology, locations of foci, types of ablation) are needed.

## Conclusion

History of hyperthyroidism increases the risk of recurrence of AF after single ablation procedure regardless of the etiological background. Higher recurrence risk was found in amiodarone-induced hyperthyroidism. Further larger prospective studies are needed to investigate the effect of different electrophysiological parameters and dose–response relationship of thyroid hormone.

## Supplementary Information

Additional file1 (DOCX 20 KB)

## Data Availability

The datasets used and/or analyzed during the current study are available from the corresponding author on reasonable request.

## Abbreviations

AF: Atrial fibrillation
AIH: Amiodarone-induced hyperthyroidism
CI: Confidence interval
CPVI: Circumferential pulmonary vein isolation
CTIA: Cavotricuspid isthmus ablation
CVD: Cardiovascular disease
HR: Hazard ration
HTH: Hyperthyroidism
LA: Left atrial
PAF: Paroxysmal AF
PeAF: Persistent AF
QUIPS: Quality in prognosis studies
RoB: Risk of bias
