# Supplementary material for: Atrial fibrillation recurrence risk after single catheter ablation in patients with history of hyperthyroidism: systematic review and meta-analysis
Source: Egypt Heart J. 2025 Oct 30;77:101. doi: 10.1186/s43044-025-00696-2 (PMC12575886; doi:10.1186/s43044-025-00696-2)
Supplement: Supplementary file 1 — Additional file1 (DOCX 20 KB) [file 43044_2025_696_MOESM1_ESM.docx]

**Supplementary materials**

**S1. Search queries**

| **Database** | **Search queries** | **Hit** |
| --- | --- | --- |
| MEDLINE (PubMED) | "ablation techniques"[MeSH Terms] AND "atrial fibrillation"[MeSH Terms] AND "hyperthyroidism"[MeSH Terms] | 11 |
| EMBASE | #1 'atrial fibrillation'  #2 'ablation therapy'/exp OR 'ablation therapy' OR 'atrial fibrillation ablation'  #3 'hyperthyroidism':ti,kw,ab  #4 #1 AND #2 AND #3 | 40 |
| Scopus | TITLE-ABS-KEY ( "ablation" )  AND  TITLE-ABS-KEY ( "atrial fibrillation"  OR  a?fib )  AND  TITLE-ABS-KEY ( hyperthyroid* ) | 154 |
| Total | | 205 |

**S2. Pre and post-ablation treatments and recorded adjusted variables**

| No. | Author, year | Primary ablation | Additional ablation | Preablation tx | Post ablation tx | Adjusted variables |
| --- | --- | --- | --- | --- | --- | --- |
| 1 | Machino, 2012[1] | CPVI | -linear ablation: left atrial roof -complex fractionated electrographic ablation -SVCI -bidirectional conduction block of cavotricuspid isthmus | -antiarrhythmic: bisoprolol, flecainide, pilsicainide, bepridil, aprindine, amiodarone -antithyroid: methimazole | -anticoagulant: oral warfarin 6 mo -antiarrhythmic drug: methimazolw, bisoprolol | history of hyperthyroidism, age (per 10 years increase), sex, AF duration (per 1 year increase), left atrial diameter (per 10 mm increase), more intensive AAD therapy prior to ablation |
| 2 | Mikhaylov, 2013[2] | CPVI | -linear ablation: roof and mitral lines with conduction block, crista terminalis -cavotricuspid isthmus ablation | -antiarrhythmic: amiodarone, other AAD -antithyroid: not specified drug, prednisone, thyroid surgery | -anticoagulant: oral warfarin 3 months, replaced by aspirin in CHADS2=0 -antiarrhythmic drugs: not specified, 3 months | NA |
| 3 | Wanwarang, 2015[3] | CPVI | -linear ablation: anterior roof, lateral mitral isthmus -complex fractionated electrographic ablation at left atrium and proximal coronary sinus -SVCI -alcoholic/ lig. of Marshall (LOM) ablation via coronary sinus -bidirectional conduction block of mitral lines | -antiarrhythmic: amiodarone, procainamide, propafenone -antithyroid drug: NA | -anticoagulant: not given -antiarrhythmic drug: not specified, 8 weeks -antithyroid drug: not given | history of hyperthyroidism, congestive heart failure, BMI, LA diameter, LA voltage, presence of non-PV ectopies |
| 4 | Wang, 2016[4] | CPVI | -linear ablation: RoC at PVI, gap at PVI, left atrial roof, mitral isthmus, tricuspid isthmus -SVCI | -anticoagulant: oral warfarin -antiarryhtmic drug: amiodarone -antithyroid drug: methimazole for severe thyrotoxicosis | -anticoagulant: oral warfarin 3 mo; extended when replase -antiarryhtmic drug: not given -antithyroid drug: methimazole for severe thyrotoxicosis | NA |

AF: atrial fibrillation; SVCI: superior vena cava isolation; PVI: pulmonary vein isolation; CPVI: circumferential pulmonary vein isolation; NA: not applicable; RoC: recovery of conduction; AAD: antiarrhythmic drug; LA: left atrial; BMI: body mass index; PV: pulmonary vein

**References**

[1] Machino T, Tada H, Sekiguchi Y, et al. Prevalence and Influence of Hyperthyroidism on the Long-Term Outcome of Catheter Ablation for Drug-Refractory Atrial Fibrillation. *Circ J* 2012; 76: 2546–2551.

[2] Mikhaylov EN, Orshanskaya VS, Lebedev AD, et al. Catheter Ablation of Paroxysmal Atrial Fibrillation in Patients with Previous Amiodarone-Induced Hyperthyroidism: A Case-Control Study: Hyperthyroidism and AF Ablation. *J Cardiovasc Electrophysiol* 2013; 24: 888–893.

[3] Wongcharoen W, Lin Y-J, Chang S-L, et al. History of hyperthyroidism and long-term outcome of catheter ablation of drug-refractory atrial fibrillation. *Heart Rhythm* 2015; 12: 1956–1962.

[4] Wang M, Cai S, Sun L, et al. Safety and efficacy of early radiofrequency catheter ablation in patients with paroxysmal atrial fibrillation complicated with amiodarone-induced thyrotoxicosis. *Cardiol J* 2016; 23: 416–421.
